# Robust and efficient representations of dynamic stimuli in hierarchical neural networks via temporal smoothing

**DOI:** 10.3389/fncom.2023.1164595

**Published:** 2023-06-15

**Authors:** Duho Sihn, Oh-Sang Kwon, Sung-Phil Kim

**Affiliations:** Department of Biomedical Engineering, Ulsan National Institute of Science and Technology, Ulsan, Republic of Korea

**Keywords:** dynamic visual motion, hierarchical structure, spatio-temporally efficient coding, robustness, smoothness

## Abstract

**Introduction:**

Efficient coding that minimizes informational redundancy of neural representations is a widely accepted neural coding principle. Despite the benefit, maximizing efficiency in neural coding can make neural representation vulnerable to random noise. One way to achieve robustness against random noise is smoothening neural responses. However, it is not clear whether the smoothness of neural responses can hold robust neural representations when dynamic stimuli are processed through a hierarchical brain structure, in which not only random noise but also systematic error due to temporal lag can be induced.

**Methods:**

In the present study, we showed that smoothness via spatio-temporally efficient coding can achieve both efficiency and robustness by effectively dealing with noise and neural delay in the visual hierarchy when processing dynamic visual stimuli.

**Results:**

The simulation results demonstrated that a hierarchical neural network whose bidirectional synaptic connections were learned through spatio-temporally efficient coding with natural scenes could elicit neural responses to visual moving bars similar to those to static bars with the identical position and orientation, indicating robust neural responses against erroneous neural information. It implies that spatio-temporally efficient coding preserves the structure of visual environments locally in the neural responses of hierarchical structures.

**Discussion:**

The present results suggest the importance of a balance between efficiency and robustness in neural coding for visual processing of dynamic stimuli across hierarchical brain structures.

## 1. Introduction

The efficient coding hypothesis, which has long been supported by researchers, suggests that information is coded in neural activity with minimal redundancy (Attneave, 1954; Barlow, 1961; Laughlin, 1981; Simoncelli and Olshausen, 2001). According to this hypothesis, each neuron codes distinct information to minimize informational redundancy. However, neural activity is intrinsically noisy, and a ubiquitous trial-to-trial variability is unavoidable (Borst and Theunissen, 1999; Faisal et al., 2008; Nogueira et al., 2020). As such, if each neuron codes distinct information maximizing efficiency, overall neural coding would be vulnerable to noise because corrupted information coded in a single neuron could not be corrected by the compensating activities of other neurons.

The vulnerability of neural coding due to the noise in a single neuron's activity can be reduced by having multiple neurons code the same information together (Chu et al., 2016; Pryluk et al., 2019). This robust coding, defined in the present study as robustness against malfunctions of brain systems, is reflected by correlations among the firing activities of a group of neurons (Montani et al., 2007; Pryluk et al., 2019). Moreover, recent studies suggest that neural coding in the brain can be characterized by the degrees of efficiency and robustness (Chu et al., 2016; Stringer et al., 2019), with an efficiency-robustness trade-off observed in various brain regions and species. For instance, a study demonstrated that efficiency is relatively more pronounced in cortical regions and humans, whereas robustness is relatively more pronounced in subcortical regions and non-human primates (Pryluk et al., 2019).

Then, how can interconnected neurons achieve both efficiency and robustness? There can be several neural coding approaches to achieve the efficiency-robustness balance. One simple approach to increase robustness while maintaining existing efficiency is to increase the number of neurons. However, this is against the energy efficiency of metabolism (Sengupta et al., 2013; Yu et al., 2016) because it increases energy consumption for processing the same information. Another possible approach is to make neural coding of information smooth such that neural representations of similar stimuli are similar (i.e., similar firing patterns of different neurons) to each other. Smooth neural representations allow for informational redundancy across neurons to some extent, gaining robustness at the expense of efficiency. A recent study demonstrates that neural coding based on smooth neural representations can elucidate the efficiency-robustness balance maintained in the visual cortex (Stringer et al., 2019).

**Figure 1:**
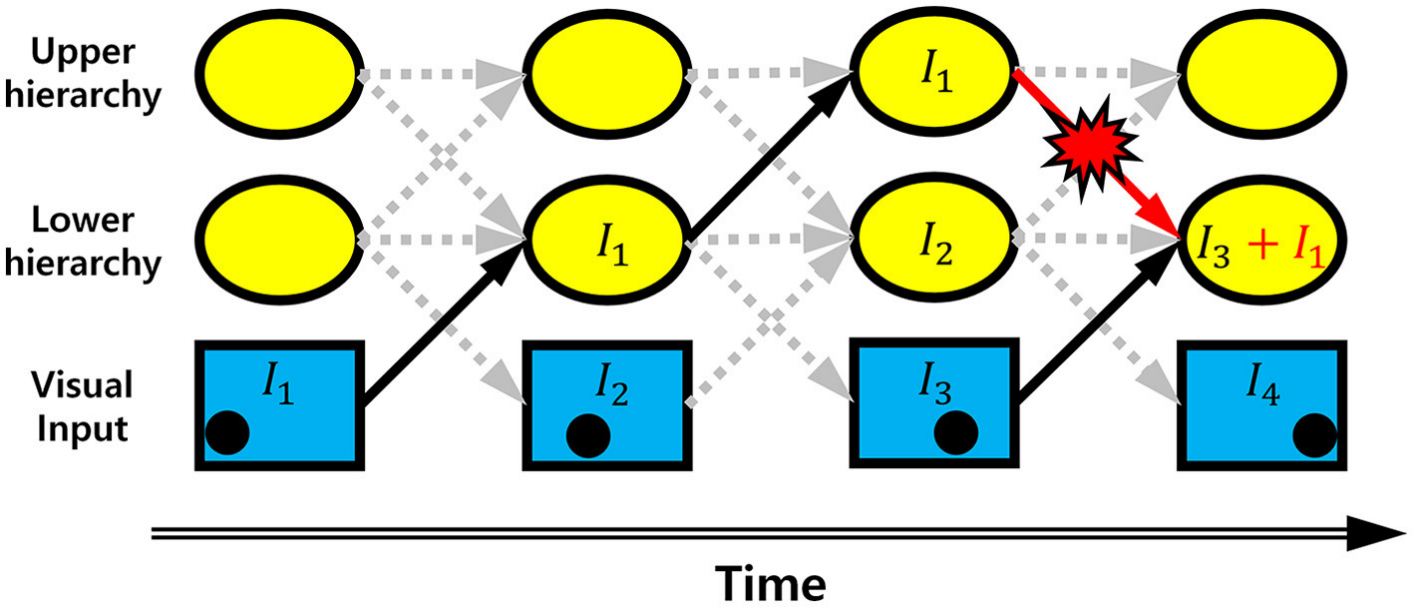
Erroneous information transmission. Schematic diagram showing the occurrence of erroneous information on a bidirectional hierarchical structure with information transmission time delay. The black dot in the bottom row indicates the position of a moving object. *I*_*t*_ is information corresponding to the position of a moving object. Arrow indicates the information transmission. As the information is transmitted through the hierarchies, the position information is delayed by one time step. If there is no erroneous information transmission, the information at the lower hierarchy of the 4th time step is *I*_3_, indicating information transmission is delayed by one time step. Due to the top-down information transmission from the upper hierarchy of 3rd time step, however, the information *I*_1_ is added to *I*_3_. This is an erroneous information.

It is well-documented that the biological visual system consists of a bidirectionally interconnected hierarchical structure (Briggs and Usrey, 2011; Harris et al., 2019; Hilgetag and Goulas, 2020; Semedo et al., 2022). Yet, it is unknown whether smooth neural representations can achieve robust neural coding when a hierarchical visual system responds to dynamic stimuli (e.g., moving objects), where neural processing of dynamic stimuli can cause a transmission delay occurring across hierarchies (Berry et al., 1999). Due to the transmission delay, when a dynamic stimulus is represented in the hierarchical visual system, an upper hierarchy carries relatively older information, whereas a lower hierarchy carries more recent information. As such, inter-hierarchy pathways continuously convey desynchronized information between hierarchies, causing errors in neural representations of stimuli at each hierarchy (Figure 1). This kind of error is not random but better regarded as systematically biased neural information delivered by desynchronized neural activities from other hierarchies. It is more likely to occur in a bidirectional hierarchical structure where neurons at each hierarchy send information to both upper and lower hierarchies simultaneously. Since the brain area processing visual information has a bidirectionally interconnected hierarchical structure (Briggs and Usrey, 2011; Harris et al., 2019; Hilgetag and Goulas, 2020; Semedo et al., 2022), it would be important to understand how the hierarchical visual system deals with the erroneous neural information when processing dynamic stimuli. With predictive coding defined on a bidirectional hierarchical structure (Rao and Ballard, 1999), it has been proposed that this problem can be solved by predicting more distant future neural responses of different hierarchies (Hogendoorn and Burkitt, 2019). However, to the best of our knowledge, no solution has been proposed to grapple with this problem with efficient coding.

In our previous study, we demonstrated that a new efficient coding model—spatio-temporally efficient coding (STEC) that adds temporal smoothing to efficient coding—enables robust neural representations of static visual images within a hierarchical visual system (Sihn and Kim, 2022). With STEC, neural responses at each visual hierarchy form smooth temporal trajectories. If the external stimulus changes to be static or smooth, smoothing the temporal trajectory of the neural responses is to make neural responses to similar stimuli similar, thus creating smooth neural representations. As STEC inherently maintains efficient neural coding by maximizing the entropy of neural activities in a population, smooth neural representations by STEC can preserve the efficiency-robustness balance by gaining robustness.

However, it remains unknown whether STEC can still hold robustness in neural representations of visually moving objects by overcoming desynchronized erroneous neural information transmitted across bidirectional visual hierarchies. While our previous study proposed a hierarchical neural network model to represent static stimuli, how a hierarchical neural network can also represent dynamic stimuli that continuously change over time becomes a challenging problem. Therefore, the present study aims to investigate whether smoothing by STEC can make neural representations of visually moving objects robust, thus maintaining the efficiency-robustness balance.

## 2. Materials and methods

### 2.1. Dataset

The natural scene image dataset created by van Hateren and van der Schaaf (1998) was used for simulations. The dataset was publicly available at http://bethgelab.org/datasets/vanhateren/. The images were downsized to 128 × 192 pixels and rescaled between zero and one. We basically used the natural scene image dataset for analysis in the previous manuscript. According to the comment, we also verified the proposed model using a different dataset of object images. We used Caltech 101 object image dataset (Li et al., 2022), which was publicly available at https://data.caltech.edu/records/mzrjq-6wc02. Since the images in this dataset vary in size, we resized them to 128 × 192 pixels. Moreover, a total of 4,212 images were randomly selected, and the pixel values were rescaled between zero and one.

### 2.2. Spatio-temporally efficient coding

**Figure 2:**
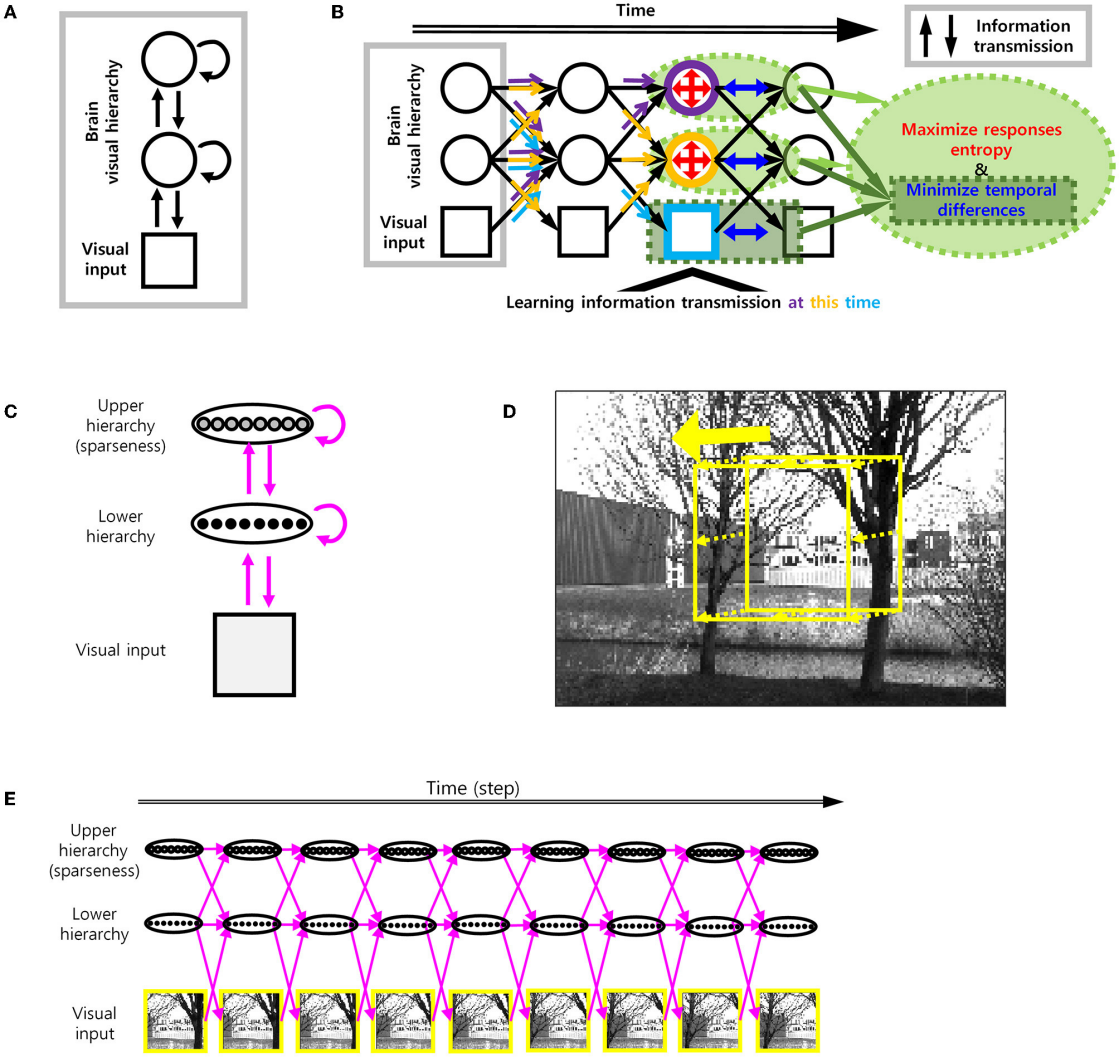
Spatio-temporally efficient coding. **(A)** The model structure of spatio-temporally efficient coding for hierarchical brain (Sihn and Kim, 2022). There are three kinds of information transmission (black arrow): Bottom-up (from lower to upper arrows), recurrent (loop arrows), top-down (from upper to lower arrows). **(B)** Spatio-temporally efficient coding (Sihn and Kim, 2022). Inference is bidirectional (bottom-up, top-down, and recurrent) information transmissions which are indicated by black arrows. These black arrows render fast neural representations through learning. Learning at each hierarchy is indicated by light blue (visual input), orange (lower hierarchy), and purple (upper hierarchy) colors. The same color-coded arrows are optimized by learning at each hierarchy. The objectives of learning are to minimize temporal differences (in visual input, lower hierarchy, and upper hierarchy; blue color) and to maximize responses entropy (in lower and upper hierarchy; red color). **(C)** Architecture of visual hierarchy with depth 2 in simulations. Units in the lower hierarchy set up to have regular neural responses and units in the higher hierarchy set up to have sparse neural responses. These correspond to lower and upper hierarchy neural responses, respectively. Units in adjacent hierarchies are fully connected. **(D)** Example movement of visual scene on the natural scene image for learning. Yellow square indicates each visual scene. **(E)** Spatio-temporally efficient coding for moving visual scene. Each yellow square corresponds to one of yellow squares in D. Magenta arrow indicates information transmission.

To add smoothness to efficient coding, we used spatio-temporally efficient coding (STEC) developed in our previous study (Sihn and Kim, 2022) with some modifications (Figures 2A, B).

The present study focuses on a hierarchical structure that consists of the visual input hierarchy, lower hierarchy, and higher hierarchy (Figure 2A). Information processing on the hierarchical structure is defined as the transformation from neural responses at each time step to neural response at the next time step over the hierarchical structure. Specifically, the information processing from time *t*−1 to *t* is a transformation such that:


(1) $$\begin{array}{l} f_{t}:X_{input,\;t-1}\times X_{h=1,t-1}\times \ldots \times X_{h=H,t-1}\to X_{input,t}\\ \times X_{h=1,t}\times \ldots \times X_{h=H,t} \end{array}$$

where *X*_*h, t*_ represents a set of all possible neural responses at hierarchy *h* and time *t* in vector form. The restriction of *f*_*t*_ to *X*_*h, t*_ can be represented as:


(2) $$\begin{array}{l} f_{t}{|}_{X_{h,t}}=\sigma \left(W_{h+1,h}^{T}x_{h+1,t-1}+W_{h,h}^{T}x_{h,t-1}+W_{h-1,h}^{T}x_{h-1,t-1}+b_{h}\right) \end{array}$$

where *x*_*h, t*−1_∈*X*_*h, t*−1_ denotes a specific neural response at hierarchy *h* and time *t*−1 in vector form, $W_{h^{\prime },h}$ denotes a synaptic weight matrix from hierarchy *h*′ to *h* and *T* denotes the transpose of a matrix, *b*_*h*_ denotes a bias term in vector form, and σ(·) denotes the sigmoid function. So *x*_*h, t*_ = *f*_*t*_|_*X*_*h, t*__∈*X*_*h, t*_ is a neural response to be used for analysis and represents the firing activity of neurons. For instance, *x*_*h, t*_ represents the vector of thenormalized firing rates of neurons located at hierarchy *h*.

Being used as a neural coding scheme on the hierarchical structure, STEC achieves efficient and robust neural representations by learning synaptic weights for the information processing above. Specifically, STEC learns weights by minimizing two objectives: *L*_*Spatial*_ and *L*_*Temporal*_. *L*_*Spatial*_ is the negative informational entropy of neural responses. The minimization of *L*_*Spatial*_ is, in fact, the objective of existing efficient coding (Barlow, 1961; Laughlin, 1981). *L*_*Temporal*_ is the difference between neural responses of adjacent time steps. The minimization *L*_*Temporal*_ renders the temporal trajectory of neural responses smooth (Figure 2B). The objective *L* of STEC is then a combination of *L*_*Spatial*_ and *L*_*Temporal*_:


(3) $$\begin{array}{l} L=L_{Temporal}+\lambda L_{Spatial} \end{array}$$

where λ is a balancing parameter. Since *L*_*Spatial*_ achieves efficiency and *L*_*Temporal*_ achieves robustness, the objective *L* of STEC can represent the efficiency-robustness balance. In the present study, we empirically set λ = 5, which maintained a balance between efficiency and robustness in neural representations. We also set λ = 1, 000 to lead STEC to be an efficient coding without the smoothness, building a control condition to be compared with balanced STEC—hereby referred to as spatially efficient coding (SEC). Finally, we set λ = 0.01 to create another condition with excessive smoothness with impoverished (spatial) efficiency—hereby referred to as temporally efficient coding (TEC).

Since *L* = *L*_*Temporal*_+λ*L*_*Spatial*_ is differentiable, the objective *L* can be minimized by the gradient descent method. In the present study, we used a stochastic gradient descent method with momentum via Adam optimizer (Kingma and Ba, 2015). The parameters of the Adam optimizer were set as α = 0.001, β_1_ = 0.9, β_2_ = 0.999, and ϵ = 10^−8^. Minimization via the Adam optimizer persisted 10^4^ iterations for one repetition. We restarted the repetition five times. For each minimization iteration above (one of 10^4^ iterations), information processing lasted on nine time steps (i.e., time step ∈[1, 9]), as we assumed that a gaze shifted 9 times in every natural scene image (Figures 2D, E). The minibatch size was set to 100.

By controlling the distribution of neural responses, we made them sparse (upper hierarchy) or non-sparse (lower hierarchy) (Figure 2C). It corresponds to non-sparse neural responses of the subcortical neurons (lower hierarchy) and sparse neural responses of the cortical neurons (upper hierarchy), respectively (Simoncelli, 2003). Neural responses of the upper hierarchy were sparse due to the following reason. The objective *L*_*Spatial*_ is the negative informational entropy of neural responses. To calculate *L*_*Spatial*_, we need a probability density function, which can be replaced with a ratio between the probability density of the neural response and the compensation density. In order to lower *L*_*Spatial*_, the probability density function should be as uniform as possible. If we increase the compensation density at near zero and at the same time make the probability density function uniform, consequently, it makes neural responses become close to 0 (i.e., sparse neural responses). In the present study, the number of neurons in each hierarchy was set to 64.

The mathematical details of STEC are described in the Supplementary material. All simulation and analysis codes for the present study are available at https://github.com/DuhoSihn/STEC_dynamic.

### 2.3. Stimuli

A moving image *X*_*input*_ was composed of a series of parts (64 × 64 pixels) of a natural scene image, where each part was obtained by moving a gaze at a constant velocity starting at a random location over the whole image (128 × 192). The magnitude of the velocity was randomly selected from an integer between 0 and 4 pixels per time step. If the selected velocity magnitude was too large to move within the whole image, the velocity magnitude was adjusted to be the maximum magnitude that allowed moving within the whole image. The direction of the velocity was randomly selected in the two-dimensional pixel space, and speed was kept as 0–4 pixels per time step with *L*_∞_ norm (the maximum of horizontal moving distance and vertical moving distance) (Figures 2C–E).

**Figure 3:**
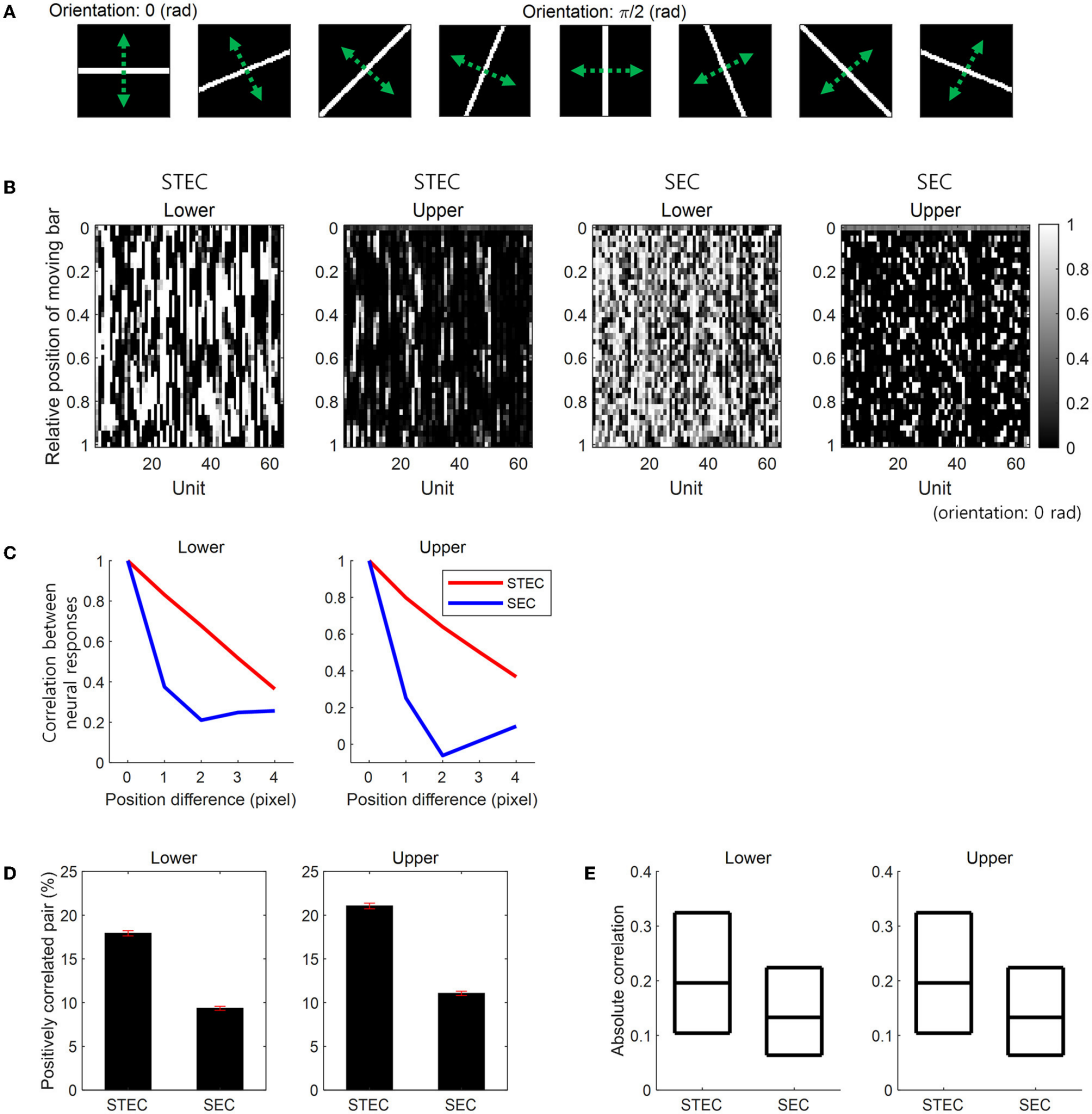
Smooth neural representations for bar stimuli. **(A)** Bar stimuli with eight orientations. **(B)** Neural responses for smoothly moving bar stimuli. Smooth changes in the vertical direction of neural responses indicate smooth neural representations. STEC indicates spatio-temporally efficient coding. SEC indicates the control condition that spatially efficient coding alone. **(C)** Correlation (over 64 units) between neural responses to moving bar stimuli with different positions. Large correlations indicate the smooth changes of neural responses in **(B)**. That is, this is a quantification of the smoothness of the neural response in **(B)**. **(D)** The ratio of statistically significant positive correlation (over time) between the temporal trajectories of the neural responses of each unit for smoothly moving bar (Pearson correlation, *p* < 0.05). Error bar indicates the SEM. **(E)** The absolute value of correlations (over time) between the temporal trajectories of the neural responses of each unit for a smoothly moving bar. Three black horizontal lines in each box indicate 25%, 50%, and 75% levels of data, respectively.

A moving bar stimulus was created as a 64 × 64 image with a value of 1 on the bar and 0 elsewhere. Bar stimuli had eight orientations: 0, π/8, 2π/8, 3π/8, 4π/8, 5π/8, 6π/8, and 7π/8 (rad). For each orientation, we created 41 bar stimuli at different positions covering the whole image uniformly. At each position, the length of a bar extended from one edge to the opposite of an image. The width of the bar was kept constant across eight orientations and occupied four pixels in the case of the horizontal orientation (Figure 3A). Over these 41 positions, we moved the bar stimulus smoothly or randomly. A smoothly moving bar in a certain orientation sequentially moved from one end position to the other end, taking 41 time steps to present a smoothly moving bar. The same bar stimulus also moved in a reversed order. For convenience, we hereafter denoted one of these bidirectional movements as moving forward and the other as moving backward, although there is no specific reason to call one as forward and the other as backward. These bidirectional movements of a bar stimulus generated a total of 16 smoothly moving bar stimuli. A randomly moving bar moved from one position to another randomly with no repeated visit to the same position, thus appearing at each of the 41 positions once and taking 41 time steps too. We generated 41 randomly moving bars in each of the eight orientations.

A static bar stimulus was the continuous presentation of one of the moving bar images at certain timing. We selected an aforementioned moving bar image at a specific point in time and continuously presented that single image with no change in time.

### 2.4. Distance between neural responses

**Figure 4:**
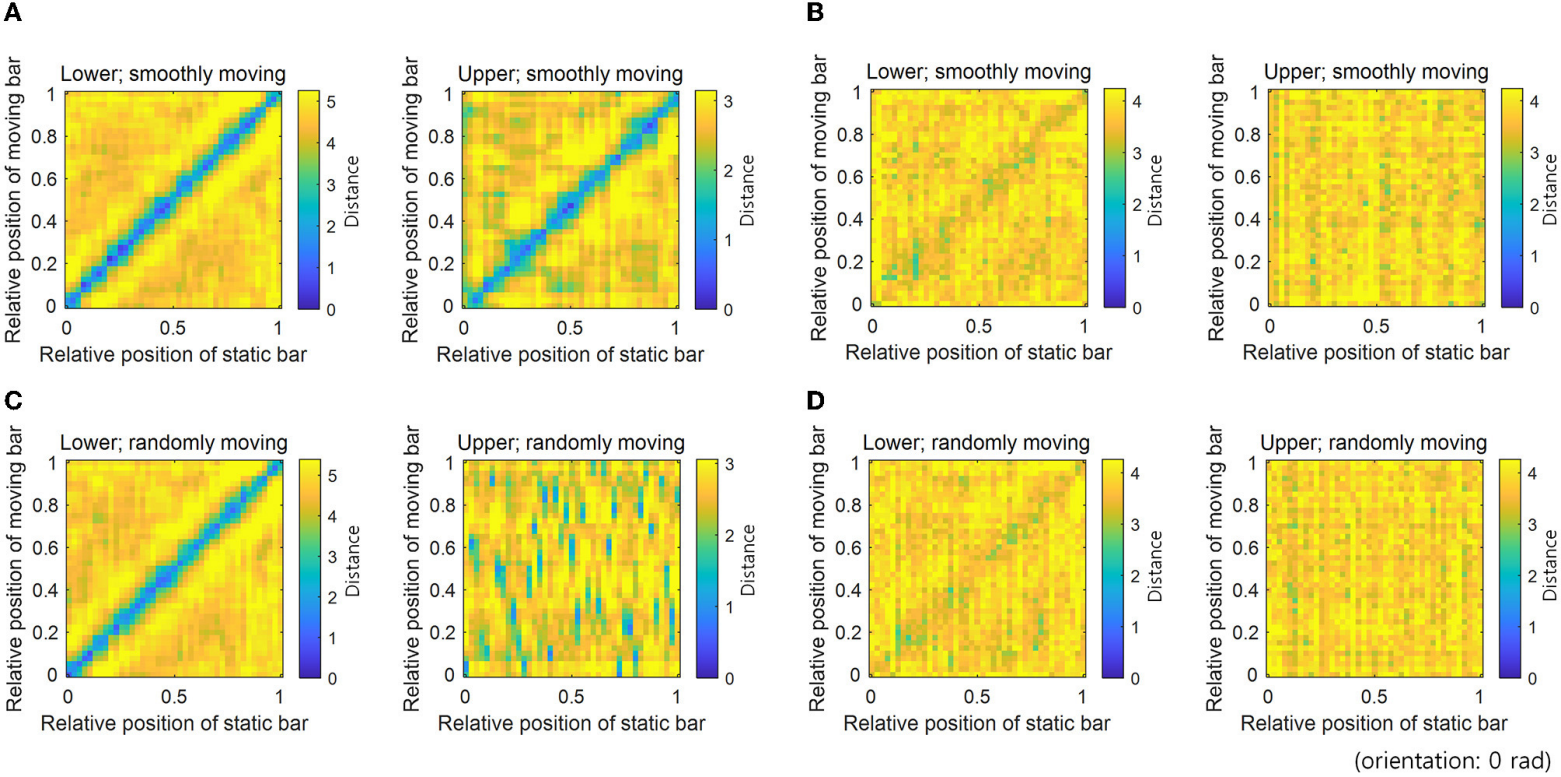
Distance between neural responses denoting robust neural representations. Distances between neural responses for static and smoothly moving bar stimuli with different positions and the 0 rad orientation: **(A)** STEC and **(B)** SEC. Distances between neural responses for static and randomly moving bar stimuli with different positions and the 0 rad orientation: **(C)** STEC and **(D)** SEC. A low distance value of diagonal entries indicates consistent neural response between static and moving bar stimuli, denoting robust neural representations. Gradually increasing distances of off-diagonal entries around diagonal entries indicate that neural responses locally preserve the structure of the external world. Consistent results were obtained for all orientations.

To measure the robustness of neural coding, we measured the Euclidean distance between the neural responses to static and moving bars in the same positions and orientations. Neural responses to a moving bar transmit erroneous information to other hierarchies. On the contrary, neural responses to a static bar do not carry such erroneous information. Therefore, neural coding is deemed to be robust to erroneous information if the distance between neural responses to static and moving bars at the same position is small. The distance between neural responses to bar stimuli (one is the static bar and another is the moving bar) with different positions and the same orientation was measured by calculating the Euclidean distance between the 64-dimensional neural response vectors (Figure 4).

### 2.5. Decoding analysis

As another method to assess the robustness of neural coding, a neural decoding model was built by using neural responses to a static bar. This decoding model was trained to estimate the information of a static bar (i.e., position or orientation) from neural responses. Then, neural responses to a smoothly moving bar were used as a test set to measure the accuracy of the trained model. Accurate decoding could signify that the neural representations of the bar stimulus for both moving and static bars are similar to each other, indicating that neural coding is deemed to be robust to erroneous information.

In the simulation of the present study, since the stimuli contained only several bars and the neural responses to the bar stimuli were deterministic, the size of neural response data for decoding was limited to training a decoding model. To augment the neural response data, random noise was added to the bar stimuli:


(4) $$\begin{array}{l} S_{\epsilon }=S+\epsilon \end{array}$$

where *S* is an original image vector, *S*_ϵ_ is the image vector with random noise, and $\epsilon \sim \vec{N\left(0,\;\sigma^{2}\right)}$ for σ = 0.025, 0.05, 0.1, 0.2, 0.4. Through this treatment, 300 different neural responses to one bar stimulus were obtained in both the training and test sets. In general, when it costs to obtain data samples, a training set includes a relatively large number of samples, and a test set includes a relatively small number of samples, in order to assign a sufficient amount of data to fully train a model. In the present study, however, we used the same size of training and test sets because it costs little to synthesize data samples. Specifically, a sample size of 300 was sufficient to train the model and another 300 samples were used for the test.

Nine centered bars in each orientation were selected to perform decoding for nine different bar positions in the same orientation. Therefore, the chance level of position decoding accuracy was 1/9. Three centered bars in each orientation were selected to perform decoding for eight different orientations. Therefore, the chance level of orientation decoding accuracy was 1/8.

**Figure 5:**
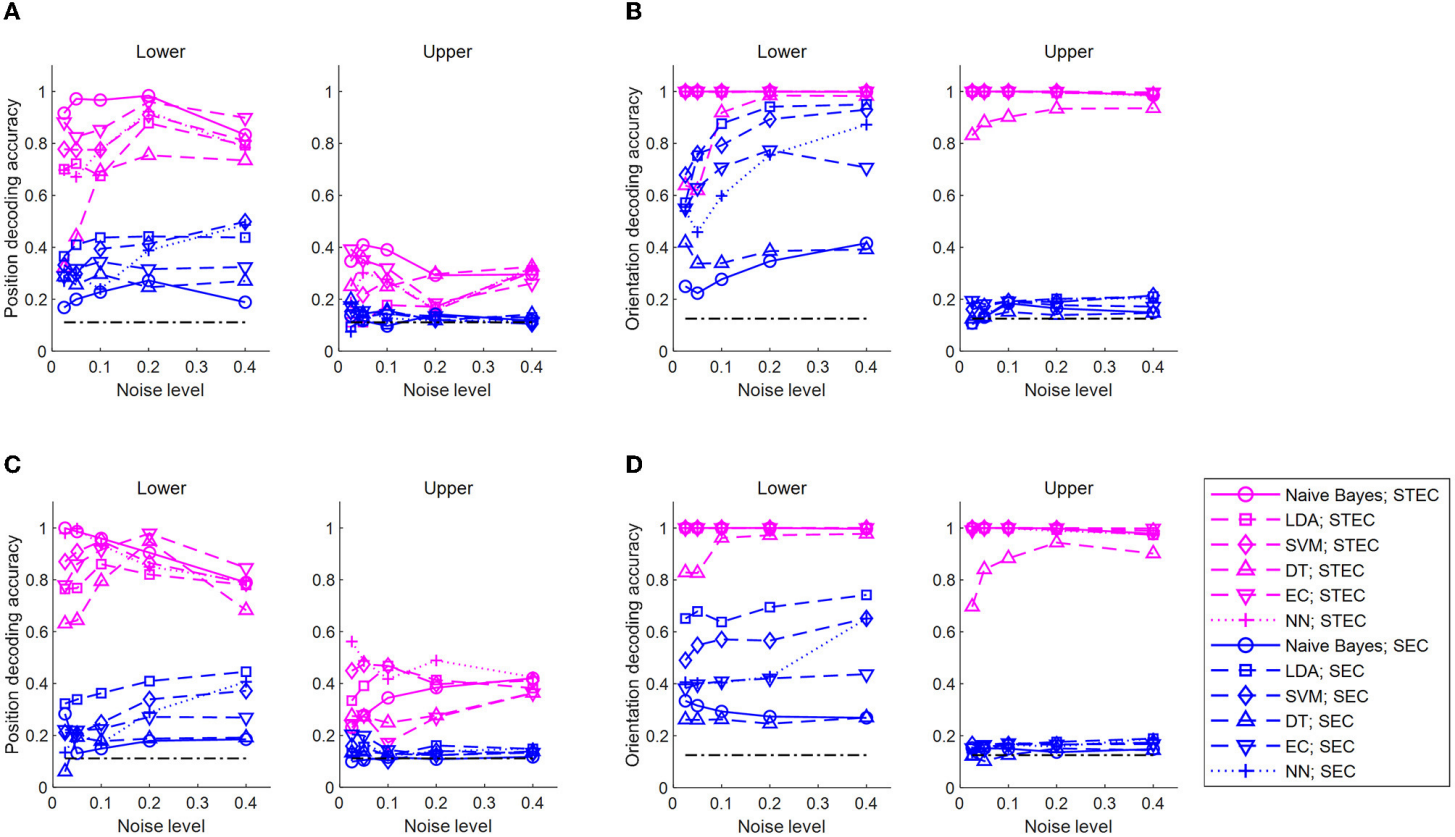
Decoding of bar stimuli denoting robust neural representations. **(A)** Position decoding accuracy at various noise levels for nine centered-bar positions. The training set is the neural responses for static bar stimuli and the test set is the neural responses for smoothly moving bar stimuli. The black dash-dotted line indicates the chance level. Natural scene dataset. **(B)** Orientation decoding accuracy at various noise levels for eight orientations of three centered bars. A training set is the neural responses for static bar stimuli and a test set is the neural responses for smoothly moving bar stimuli. The black dash-dotted line indicates the chance level. Natural scene dataset. **(C)** Similar to A, but object dataset. **(D)** Similar to **(B)**, but object dataset. A high accuracy value indicates consistent neural response between static and moving bar stimuli, suggesting robust neural representations. Noise levels are the standard deviations of the added random noise (see Materials and Methods). Decoding is performed with a naïve Bayes classifier, linear discriminant analysis (LDA), support vector machine (SVM), decision tree (DT), ensemble classifier, and 5-layers neural network (NN).

Decoders with two different characteristics were used. For one, the naïve Bayes, which decodes by estimating the mean and variance of the data, was selected. Another was linear discriminant analysis (LDA), which decodes data without estimating the distribution of the data. We additionally applied a support vector machine (SVM) with linear kernel, decision tree (DT), and ensemble classifier (EC) for decoding to compare machine learning techniques more comprehensively. In addition, a 5-layer neural network (NN) was used for decoding to investigate the feasibility of using a basic deep learning technique (see Figure 5).

## 3. Results

### 3.1. Smooth neural representations for bar stimuli via spatio-temporally efficient coding

We compared STEC to a control condition with spatially efficient coding (SEC) only. SEC was implemented by amplifying *L*_*Temporal*_ in STEC. First, we examined whether STEC generated smooth neural representations. We observed that the neural responses to a smoothly moving bar changed smoothly in both lower and upper hierarchies. In contrast, with SEC, the change of the neural responses to the smoothly moving bar was not smooth (Figure 3B). To quantify the smoothness of neural responses, we calculated pairwise correlations among the neural responses of 64 units to two moving bar stimuli at different but adjacent positions. Since the bar moves smoothly, the neural responses to an adjacent bar are temporally adjacent neural responses. Therefore, higher correlations between neural responses to adjacent bars indicate temporally more similar neural responses, meaning smoother trajectories of neural responses. STEC-generated neural responses showed higher correlations than SEC-generated neural responses (Figure 3C). This suggests that SEC exhibited higher efficiency.

Previous studies on smoothness evaluated robustness against random noise (Stringer et al., 2019) by verifying correlated activity between neuronal units (Montani et al., 2007; Pryluk et al., 2019). Therefore, we also evaluated whether activities between neuronal units were correlated in the presence of smoothness induced by spatio-temporally efficient coding. The correlation between the temporal trajectories of the neural responses of each unit to a smoothly moving bar in each orientation was calculated. With STEC, there were more statistically significant (Pearson correlation, *p* < 0.05) positively correlated pairs of neuronal units (Figure 3D) than SEC (rank-sum test, *p* = 2.7 × 10^−4^ and *p* = 7.2 × 10^−87^, for lower and upper hierarchies, respectively). Also, the absolute value of correlations was higher with STEC than with SEC (Figure 3E) (rank-sum test, *p*≈0 and *p* = 1.6 × 10^−63^, for lower and upper hierarchies, respectively).

### 3.2. Robust neural representations via smoothness

The distance between neural responses for static and moving bars in the same position was small in both lower and upper hierarchies with STEC (Figure 4A), compared to that with SEC (Figure 4B), indicating robust neural coding against erroneous information by STEC. In addition, distances in off-diagonal entries around diagonal entries increased gradually, indicating that neural responses locally preserve the structure of the external world (Figure 4A). In contrast, the neural responses in the upper hierarchy to a randomly moving bar were no longer smooth, showing that the neural coding was not robust to erroneous information when a stimulus was not smoothly moving (Figure 4C).

Decoding the position of a bar resulted in higher accuracy with STEC than with SEC in lower and upper hierarchies, regardless of decoder type (Figures 5A, C). Decoding the orientation of a bar resulted in higher accuracy with STEC than with SEC regardless of the decoder type (Figures 5B, D).

### 3.3. The balance between efficiency and robustness

**Figure 6:**
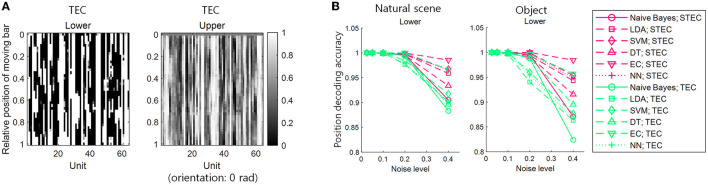
The importance of a balance between efficiency and robustness. **(A)** Neural responses for smoothly moving bar stimuli. TEC indicates temporally efficient coding, which exhibits excessive smoothness to achieve excessive robustness and lack efficiency. **(B)** Position decoding accuracy at various noise levels for nine centered-bar positions. Both training and test sets are the neural responses for smoothly moving bar stimuli. Noise levels are the standard deviations of the added random noise (see Materials and Methods). Decoding is performed with a naïve Bayes classifier, linear discriminant analysis (LDA), support vector machine (SVM), decision tree (DT), ensemble classifier, and 5-layers neural network (NN).

We denoted TEC as the condition that exhibits excessive smoothness to achieve robustness with the lack of efficiency. In this condition, the neural responses to a smoothly moving bar changed too smoothly to discriminate neural responses to adjacent bars in both lower and upper hierarchies (Figure 6A). This may lead to indistinguishable neural responses to different stimuli. To confirm this, a position decoding analysis was performed using neural responses to smoothly moving bars on both the training and test sets. The details of the position decoding were identical to those in the previous subsection (Figure 5A, see also Materials and Methods section), except for using neural responses to smoothly moving bars in both the training and test sets. The decoding result showed that the position decoding accuracy was lower in TEC than in STEC, indicating that excessive robustness led to less distinguishable neural responses to different stimuli (Figure 6B). This result suggests that a balance between efficiency and robustness is important.

When the coding objective has only spatial efficiency with no temporal efficiency (i.e., SEC), the efficiency increases, but the robustness decreases (Figures 4, 5). Conversely, when the coding objective has only temporal efficiency with no spatial efficiency (i.e., TEC), the redundancy of neural coding is too large to achieve the required efficiency (Figure 6). Therefore, when it is necessary to emphasize brain efficiency by reducing redundancy, it is better to emphasize the spatial efficiency of the STEC more, and when it is necessary to emphasize robustness, it is better to emphasize the temporal efficiency of the STEC more. This may elucidate the actual efficiency-robustness trade-off observed across brain regions or species (Pryluk et al., 2019).

## 4. Discussion

It is known that efficiency and robustness are in balance in neural activity. To understand how the brain maintains such a balance, it is useful to employ a computational model to emulate the brain functions with both efficiency and robustness. Efficient coding has been proposed as a neural coding principle to achieve efficiency, defined as minimizing informational redundancy. Also, the smoothness of neural representations is known to improve robustness (defined in the present study as robustness against malfunctions of brain systems) against random noise (Stringer et al., 2019). However, in the case when erroneous neural information is transmitted across the brain hierarchy due to dynamic stimuli, it is unknown whether smoothness can sustain the robustness of neural representations for dynamic stimuli. In the present study, we showed that spatio-temporally efficient coding (Sihn and Kim, 2022) could achieve robustness against erroneous neural information. As a neural coding model is used to implement efficiency, our results suggest that spatio-temporally efficient coding could generate both efficient and robust neural representations over a hierarchical structure.

Spatio-temporally efficient coding minimizes temporal differences between the present and future neural responses. It thereby renders the temporal trajectories of neural responses smooth. Smoothly changing external stimuli generate smooth neural representations in the brain via spatio-temporally efficient coding. This was also shown in the present study using bar stimuli (Figures 3B, C) which are frequently selected as experimental stimuli in studies on early visual systems (Hubel and Wiesel, 1968; Williams et al., 2021; Summers and Feller, 2022). It also produced correlated activity between units, representing robustness to random noise (Figures 3D, E).

Neural responses to moving bars in the brain hierarchy transmit erroneous information to other hierarchies. On the other hand, neural responses to static bars do not have such erroneous information. Therefore, if the neural responses to static bars and moving bars are similar, it can be said that this neural coding is robust to erroneous information by reducing the degree of the error. This was shown through the distance between neural responses (Figure 4) and the decoding of bar stimuli (Figure 5) in our simulations, indicating that smooth coding via spatio-temporally efficient coding reduces the impact of erroneous information.

This robustness could be achieved because spatio-temporally efficient coding is smooth coding that preserves structures of the external world locally in the brain. When the external world changes smoothly, smooth coding, which reflects the smooth change in neural response, preserves the difference in the appearance of the external world as a difference in neural response. This was also confirmed in the simulations in the present study; gradually increasing distances of off-diagonal entries around diagonal entries indicate that neural responses locally preserve the structure of the external world (Figure 4A). If the local structure of the external world is preserved in the neural response and the time difference between the present and the past is not very large, a similar appearance of the external world will be reflected in the similar neural response. These similar neural responses between the present and the past will reduce the difference in information represented in different hierarchies due to transmission delay. This means that the impact of erroneous information is reduced.

Reducing the difference in information represented between the present and the past also means that the difference in information represented between the present and the future is decreasing. This is consistent with the predictive information that the biological visual system has information about future stimuli in advance (Palmer et al., 2015; Chen et al., 2017; Sederberg et al., 2018; Liu et al., 2021). It is known that a biologically plausible receptive field can be obtained by using such future predictive coding (Singer et al., 2018). The efficient coding principle for future prediction through the information bottleneck framework was also presented (Chalk et al., 2018).

It is known that sustained eye movements play an important role in visual information processing to grasp detailed information (Rucci and Poletti, 2015; Rucci and Victor, 2015). Since these eye movements substantially alter visual input, rapid stabilization of neural responses in the visual hierarchy is required to process such continuously changing visual information. A recent study has shown that the early visual system primarily responds to rapidly changing visual input and that adjacent neurons exhibit similar responses, indicating smooth neural representation (Schottdorf and Lee, 2021). This is consistent with the smooth neural representation in neural network models learned with STEC in the present study.

Recent research has shown that correlations between the responses of neurons in the visual system become stronger as the hierarchy increases (Siegle et al., 2021). These correlated neural responses were also predicted by a computational model learned through the efficient coding of static images (Kong et al., 2018). These results are consistent with the correlated neural responses observed over the hierarchy in our model. Recent efficient coding studies have revealed that efficient information transfer is achieved even between hierarchical structures (Zhou et al., 2022). The temporally smoothed neural responses implemented in our model can reduce unnecessary fluctuations in information transmission between hierarchical structures, thus enabling efficient information transmission. Although our study is limited to visual hierarchy, it would be plausible to extend STEC to other brain hierarchies as a recent predictive coding model has demonstrated experimental results on the whole brain hierarchy (Chao et al., 2018).

In the simulation of this study, neural representations for visual bar stimuli were investigated. However, there is a possibility that the same conclusion can be reached for other changing visual or sensory stimuli. The first limitation of this study is the lack of extensive investigation of neural representations for different kinds of dynamic environments. The second limitation is that the low decoding accuracy in the control condition (SEC) appears not only in static (training set)–moving (test set) bar decoding but also in static–static bar decoding and moving–moving bar decoding (data was not shown). The low decoding performance under these control conditions (SEC) was predicted in previous studies as well (Sihn and Kim, 2022). For a more precise argument, it is necessary to verify a control condition of static–static bar decoding or moving–moving bar decoding. The third limitation is that the model in the present study was insufficient to explain various visual perceptual phenomena induced by dynamic visual stimuli. The current hierarchical structure is too simple to elucidate various visual perceptual phenomena, including motion perception in the higher hierarchy. The motion information of the higher hierarchy of the real brain can differentiate the neural representations of the static bar and the moving bar (against Figures 4, 5). However, since spatio-temporally efficient coding can be readily applied to deeper hierarchy with little modification, it will be plausible to extend spatio-temporally efficient coding to more complex visual hierarchical structures and explore how the known properties of visual perception emerge from neural responses.

## 5. Conclusion

While the efficient coding hypothesis that minimizes redundancy has long been accepted, it is susceptible to brain malfunctions such as noise. Balanced robustness and efficiency of neural representations of external stimuli are observed in biological neuronal activities and are presumably a key aspect of neural coding. Efficient neural representations can be formed by minimizing information redundancy among neurons and robust neural representations can be formed by making neural responses smooth. However, when these neural coding schemes are applied to a hierarchical structure with bidirectional signal pathways in the brain (e.g., visual hierarchy), they encounter a problem of dealing with erroneous neural information due to transmission delays across hierarchies. The problem emerges more clearly when representing dynamic stimuli that change over time. The present study aims to deal with this problem by using spatio-temporally efficient coding (STEC), which can generate temporally smooth neural representations of dynamic stimuli. Temporal smoothing, in particular, enables smooth neural representations of dynamic stimuli that change smoothly over time. Such temporally smooth neural representations mitigate the problem of transmitting erroneous neural information across hierarchies, resulting in robust neural representations. The present study may help to deepen our understanding of how neurons in a hierarchical structure in the brain efficiently code the information of dynamic stimuli by maintaining the robustness–efficiency balance.

## Data availability statement

The original contributions presented in the study are included in the article/Supplementary material, further inquiries can be directed to the corresponding authors.

## Author contributions

DS: conceptualization, methodology, software, validation, formal analysis, investigation, resources, data curation, writing—original draft, writing—review and editing, visualization, supervision, and project administration. O-SW: writing—original draft, writing—review and editing, visualization, and supervision. S-PK: writing—original draft, writing—review and editing, visualization, supervision, project administration, and funding acquisition. All authors contributed to the article and approved the submitted version.
