# Supplementary material for: Robust and efficient representations of dynamic stimuli in hierarchical neural networks via temporal smoothing
Source: Front Comput Neurosci. 2023 Jun 15;17:1164595. doi: 10.3389/fncom.2023.1164595 (PMC10307978; doi:10.3389/fncom.2023.1164595)
Supplement: Supplementary file 1 [file Data_Sheet_1.docx]

Supplementary Material

Robust and efficient representations of dynamic stimuli in hierarchical neural networks via temporal smoothing

Duho Sihn, Oh-Sang Kwon^*^, Sung-Phil Kim^*^

*** Correspondence:** Sung-Phil Kim: [spkim@unist.ac.kr](mailto:spkim@unist.ac.kr), Oh-Sang Kwon: [oskwon@unist.ac.kr](mailto:oskwon@unist.ac.kr).

# Implementation of spatio-temporally efficient coding

Below, we describe spatio-temporally efficient coding again as introduced in Sihn and Kim (Sihn and Kim, 2022).

Spatio-temporally efficient coding aims to model the efficient use of given resources by neural systems. Spatio-temporally efficient coding can be defined on a hierarchical structure, $X_{\mathrm{input}}\times X_{h=1}\times\ldots\times X_{h=H}$ where $X_{\mathrm{input}}$ denotes a visual input and $X_{h=j}$ denotes neural responses at the *j*-th brain hierarchy. Spatio-temporally efficient coding involves the transmission of information bidirectionally (bottom-up and top-down) and recurrently on the hierarchy (Fig. 2A).

Information processing in spatio-temporally efficient coding (i.e., information transmission across a hierarchical structure) is defined as a function $f_{t}$ from time $t-1$ to time $t$ such that:

$$\begin{matrix} f_{t}:X_{\mathrm{input}, t-1}\times X_{h=1, t-1}\times\ldots\times X_{h=H, t-1}\to X_{\mathrm{input}, t}\times X_{h=1, t}\times\ldots\times X_{h=H, t} & (S1) \end{matrix}$$

, where $X_{h,t}$ denotes a vector space of neural responses in each hierarchy $h>0$ at time $t$ and $X_{\mathrm{input}, t}$ denotes a vector space of visual inputs at time $t$. If we let $X_{h=0, t}$ represent $X_{\mathrm{input}, t}$, then $f_{t}$ is given by

$$\begin{matrix} f_{t}\left. \right|_{X_{h,t}}=\sigma\left( W_{h+1,h}^{T}x_{h+1,t-1}+W_{h,h}^{T}x_{h,t-1}+W_{h-1,h}^{T}x_{h-1,t-1}+b_{h} \right) & (S2) \end{matrix}$$

, where $0\leq h\leq H$. $f_{t}\left( \cdot\right)\left. \right|_{X_{h,t}}$ represents restricting the range of the function value, $f_{t}$, to $X_{h,t}$. $W_{h+1,h}$ is a weight matrix from hierarchy $h+1$ to $h$, $T$ is the transpose of a matrix, $x_{h+1,t-1}\in X_{h+1,t-1}$is a vector of specific neural responses at hierarchy $h+1$ and time *t*-1, $b_{h}$ is a bias vector at hierarchy $h$, and $\sigma(\cdot)$ is a component-wise sigmoid function. When $h+1>H$, the term of $W_{h+1,h}^{T}x_{h+1,t-1}$ would be omitted. When $h-1<0$, the term of $W_{h-1,h}^{T}x_{h-1,t-1}$ and $W_{h,h}^{T}x_{h,t-1}$ would be omitted.

Learning in spatio-temporally efficient coding is to minimize the two objectives, $L_{Temporal}$ and $L_{Spatial}$, to efficiently use given time and space resources (Fig. 2B). The first objective, $L_{Temporal}$ of temporally efficient coding, is given by

$$\begin{matrix} L_{Temporal}=\sum_{n=1}^{N} \left( f_{t}\left( \tilde{x}_{\vec{h},t-1,n} \right)-f_{t+1}\left( \tilde{x}_{\vec{h},t,n} \right) \right)^{2} & (S3) \end{matrix}$$

where 2-square-operation is component-wise, $n$ indicates an index of a dynamic visual scene (movie), $N$ indicates the number of dynamic visual scenes (movies) used for simultaneous learning (i.e., $N$ is the minibatch size), and $\tilde{x}_{\vec{h},t-1,n}$ indicates neural responses and visual input (image) of $n$th dynamic visual scene (movie) at time $t-1$. $f_{t}\left( \tilde{x}_{\vec{h},t-1,n} \right)=\tilde{x}_{\vec{h},t,n}$ indicates the present neural responses, and $f_{t+1}\left( \tilde{x}_{\vec{h},t,n} \right)=\tilde{x}_{\vec{h},t+1,n}$ indicates the future neural responses. Minimizing temporal differences between the present and future neural responses (i.e., minimization of $L_{Temporal}$) allows the efficient use of given time resources by generating neural representations of visual inputs as quickly as possible. In addition, minimizing temporal differences in neural responses renders the temporal trajectories of neural responses smooth. In other words, when an external stimulus changes smoothly over time such that consecutive stimuli are not much different from each other, the corresponding neural responses would also be similar to each other due to temporal smoothness.

However, there is a trivial solution for the minimization of temporal differences in neural responses: Neural responses are not changed regardless of the dynamics of external visual stimuli. To avoid this problem, we need the second objective, $L_{Spatial}$ of spatially efficient coding, given by:

$$\begin{matrix} L_{Spatial}=\sum_{n=1}^{N} \log P\left( f_{t}\left. \right|_{X_{h}}\left( \tilde{x}_{\vec{h},t-1,n} \right) \right) & (S4) \end{matrix}$$

, where $P\left( \cdot\right)$ is a probability. Since $L_{Spatial}$ represents a negative informational entropy, minimization of $L_{Spatial}$ maximizes the neural response range of $f_{t}$. Also, minimizing $L_{Spatial}$ increases differences in neural responses to distinct visual stimuli, allowing neural responses to change with dynamically changing external visual stimuli. In fact, minimizing $L_{Spatial}$ is the objective of the existing efficient coding (Barlow, 1961; Laughlin, 1981).

To minimize $L_{Spatial}$, the calculation of the probability, $P\left( f_{t}\left. \right|_{X_{h}} \right)$ should precede. To obtain the exact form of a probability density, we need to calculate the partition function. However, calculating the partition function is challenging, and an alternative solution is to minimize $L_{Spatial}$ without calculating the exact $P\left( f_{t}\left. \right|_{X_{h}} \right)$ by using a pre-normalized density based on the sample density. This pre-normalized density is computed by using the kernel density estimation with a Gaussian kernel (We set the width of the Gaussian kernel as $0.8=0.1 \left( \dim X_{h} \right)^{1/2}$ where $\dim X_{h}=64$.) as follows:

$$\begin{matrix} P\left( f_{t}\left. \right|_{X_{h}} \right)=\frac{Q\left( f_{t}\left. \right|_{X_{h}} \right)}{Q'\left( f_{t}\left. \right|_{X_{h}} \right)} & (S5) \end{matrix}$$

, where $Q\left( f_{t}\left. \right|_{X_{h}} \right)$ is a neural response density and $Q'\left( f_{t}\left. \right|_{X_{h}} \right)$ is a compensation density. The neural response density $Q\left( f_{t}\left. \right|_{X_{h}} \right)$ is obtained by the kernel density estimation. The compensation density $Q'\left( f_{t}\left. \right|_{X_{h}} \right)$ measures the density of pseudo-uniformly generated samples on $X_{h}'$ (sample generating space) instead of neural responses, in order to compensate for the non-uniformity of $Q\left( \cdot\right)$ on a bounded space via the same type of non-uniformity of $Q'\left( \cdot\right)$. Typically, $X_{h}'=X_{h}=\left[ 0, 1 \right]^{\dim X_{h}}$ where $\dim X_{h}$ denotes the dimension of $X_{h}$. However, when we want to add a condition of sparse neural responses, we can set $X_{h}'=\left[ -1, 1 \right]^{\dim X_{h}}$. Such setting makes kernel density $Q'\left( f_{t}\left. \right|_{X_{h}} \right)$ to be estimated more often nearby 0, and consequently leads $P\left( f_{t}\left. \right|_{X_{h}} \right)$ around 0 to be smaller. Then, minimizing negative entropy will increase $Q\left( f_{t}\left. \right|_{X_{h}} \right)$ nearby 0 so as to maintain $P\left( f_{t}\left. \right|_{X_{h}} \right)$ close to uniform distribution. The increased $Q\left( f_{t}\left. \right|_{X_{h}} \right)$ nearby 0 means that many neural responses reduce to 0, leading to sparse neural responses. In our simulation of the present study, $X_{h}'=X_{h}=\left[ 0, 1 \right]^{\dim X_{h}}$ when $h=1$ and $X_{h}'=\left[ -1, 1 \right]^{\dim X_{h}}$ when $h=2$ (Fig. 2C). It corresponds to non-sparse neural responses of the subcortical neurons ($h=1$; lower hierarchy) and sparse neural responses of the cortical neurons ($h=2$; upper hierarchy), respectively (Simoncelli, 2003). In the present study, the number of neurons ($=\dim X_{h}$) in each hierarchy was set to 64.

The objective of spatio-temporally efficient coding is minimizing both $L_{Temporal}$ and $L_{Spatial}$:

$$\begin{matrix} L=L_{Temporal}+\lambda L_{Spatial} & (S6) \end{matrix}$$

where $\lambda$ is a balancing parameter. In spatio-temporally efficient coding, $L_{Temporal}$ and $L_{Spatial}$ must be properly balanced. In our simulation of the present study, we set $\lambda=5$ for spatio-temporally efficient coding, and denote it as STEC (spatio-temporally efficient coding). The control condition is an efficient coding without the smoothness. In our simulation of the present study, we set $\lambda=1000$ for the control condition, thereby relatively reducing the smoothness. We denote it as SEC (spatially efficient coding). Lastly, another condition is the excessive smoothness which lacks (spatial) efficiency. In our simulation of the present study, we set $\lambda=0.1$ for such a condition. We denote it as TEC (temporally efficient coding).

Since $L=L_{Temporal}+\lambda L_{Spatial}$ is differentiable, the objective $L$ can be minimized by the gradient descent method. In the present study, we used a stochastic gradient descent method with momentum via Adam optimizer (Kingma and Ba, 2015). The parameters of Adam optimizer were set as $\alpha=0.001$, $\beta_{1}=0.9$, $\beta_{2}=0.999$, and $\epsilon={10}^{-8}$. Minimization via Adam optimizer persisted ${10}^{4}$ iterations per one repetition. We restarted the repetition five times. For each minimization iteration above (one of ${10}^{4}$ iterations), $f_{t}$ lasted on nine-time steps (i.e., $t\in\left[ 1,9 \right]$), as we assumed that a gaze shifted 9 times in every natural scene image (Fig. 2D and 2E). The minibatch size was set to 100.

# References

1. Barlow HB. Possible principles underlying the transformations of sensory messages. In: Rosenblith WA editor. Sensory Communication, MIT Press; 1961. p 217-234. doi: 10.7551/mitpress/9780262518420.003.0013
2. Kingma DP and Ba J. Adam: A method for stochastic optimization. Proceedings of the 3th International Conference on Learning Representations; 2015 May 7-9; San Diego, California, United States.
3. Laughlin SB. A simple coding procedure enhances a neuron's information capacity. Z. Naturforsch. 1981; 36(9-10):910-912 doi: 10.1515/znc-1981-9-1040
4. Sihn D and Kim S-P. Spatio-temporally efficient coding assigns functions to hierarchical structures of the visual system. Front. Comput. Neurosci. 2022 May 27; 16:890447. doi: 10.3389/fncom.2022.890447
5. Simoncelli EP. Vision and the statistics of the visual environment. Current Opinion in Neurobiology 2003 Apr 01; 13(2):144-149. doi: 10.1016/S0959-4388(03)00047-3
